# Association between first-year axial elongation and 10-year myopia progression in children wearing orthokeratology lenses: a ten-year longitudinal study

**DOI:** 10.3389/fcell.2026.1786621

**Published:** 2026-03-13

**Authors:** Zixun Wang, Xin Liu, Xiaoling Zhang, Boxuan Sun, Jinghui Wang, Xiaoxue Hu, Zhiqing Li, Weiping Lin, Bei Du, Ruihua Wei

**Affiliations:** 1 Tianjin Key Laboratory of Retinal Functions and Diseases, Tianjin Branch of National Clinical Research Center for Ocular Disease, Eye Institute and School of Optometry, Tianjin Medical University Eye Hospital, Tianjin, China; 2 Handan Eye Hospital (The Third Hospital of Handan), Handan, Hebei, China; 3 Wuhan Children’s Hospital (Wuhan Maternal and Child Healthcare Hospital), Tongji Medical College, Huazhong University of Science & Technology, Wuhan, Hubei, China

**Keywords:** axial length, first-year progression, long-term efficacy, nomogram, orthokeratology, predictive model

## Abstract

**Objective:**

To investigate the correlation between first-year axial elongation and 10-year (adulthood) axial length (AL) outcomes in children wearing orthokeratology (Ortho-K) lenses, and to develop predictive models for long-term myopia control.

**Methods:**

Data from 101 children who wore Ortho-K lenses for 10 years (2011–2025 cohort) were analyzed. Feature selection was performed using Single- and multi-factor logistic regression, LASSO, and the Boruta algorithm. Outcomes included AL > 26 mm after 10 years and rapid late-stage progression (annual increase >0.1 mm between years 9 and 10). Predictive models were visualized via dynamic nomograms.

**Results:**

First-year AL change (Change AL), baseline AL, and flat eccentricity (Flat E) were significant predictors for 10-year AL > 26 mm. The optimized model achieved an AUC of 0.952. A model only combining Chang AL predicted progression in years 9–10 with an AUC of 0.995. The optimal Chang AL cutoff for predicting stable 10-year progression was 0.28 mm overall (AUC = 0.978). Age-specific cutoffs were 0.28 mm for 8–9 years and 0.29 mm for 10–13 years.

**Conclusion:**

The first-year treatment response is an important indicator of adult-stage and long-term myopia progression in Ortho-K wearers. Monitoring the 0.28 mm first-year elongation threshold enables early identification of children at risk of poor long-term control.

## Introduction

Myopia has become a significant global public health issue (Chen et al., 2023) (Naidoo et al., 2019) (Yam et al., 2025). Latest data indicate that by 2050, the prevalence of myopia among children will exceed 50% both in Asia and globally (Pan et al., 2025) (Pärssinen and Grzybowski, 2025). This trend will be accompanied by increased rates of high myopia (HM) and pathological myopia (PM) in adulthood, ultimately leading to irreversible vision impairment (Yang et al., 2025) (Han et al., 2022). Axial length (AL) is a significant biomechanical factor contributing to the development of PM. As AL increases, retinal pathologies can lead to severe vision loss and substantial economic burden (Bullimore et al., 2021; Dai J. et al., 2025).

One key indicator of myopia control is AL progression (Li et al., 2025). Studies indicate that as AL progresses, the probability of developing PM and visual field defects also increases (Jiang et al., 2023) (Jonas et al., 2025). Although myopia and AL tend to stabilize with age, the latest research indicates that further progression of AL remains possible even in adulthood (Wang et al., 2025) (Whayeb et al., 2025). Research suggests that controlling myopia in children is an effective measure to reduce the future incidence of HM (Jonas et al., 2021) (Yii et al., 2024) (Grzybowski et al., 2020). AL exceeding approximately 26 mm has been consistently associated with a substantially increased risk of myopic maculopathy, optic nerve damage, and irreversible visual impairment, and is therefore commonly used as a surrogate structural risk marker in longitudinal myopia studies.

In recent years, myopia management has gradually shifted from vision correction to myopia control (Bullimore et al., 2025). Certain myopia prevention and control methods are gradually being incorporated into myopia management treatment protocols. Examples include red light therapy, defocus-inducing spectacle frames, and low-concentration atropine eye drops (Hu et al., 2026) (Zhang et al., 2024) (Han et al., 2023). Orthokeratology (Ortho-K) lenses, a classic method for controlling myopia and AL progression, have demonstrated short-term efficacy (Zaabaar et al., 2025) (Han et al., 2025). As treatment progresses, there are currently few reports on the progression of AL and the ultimate resolution of AL in adolescents who persist with long-term Ortho-K lenses wear into adulthood. At the same time, it remains unclear whether the baseline refractive data and early AL changes associated with Ortho-K lenses hold predictive value for the ultimate effectiveness of myopia control.

Therefore, this study collected refractive information from children who had worn Ortho-K lenses for up to 10 years. The objective was to investigate the influence and predictive value of baseline Ortho-K lens wear data and early AL changes on long-term AL outcomes and the stability of progression into adulthood.

## Methods

### Study design

**FIGURE 1:**
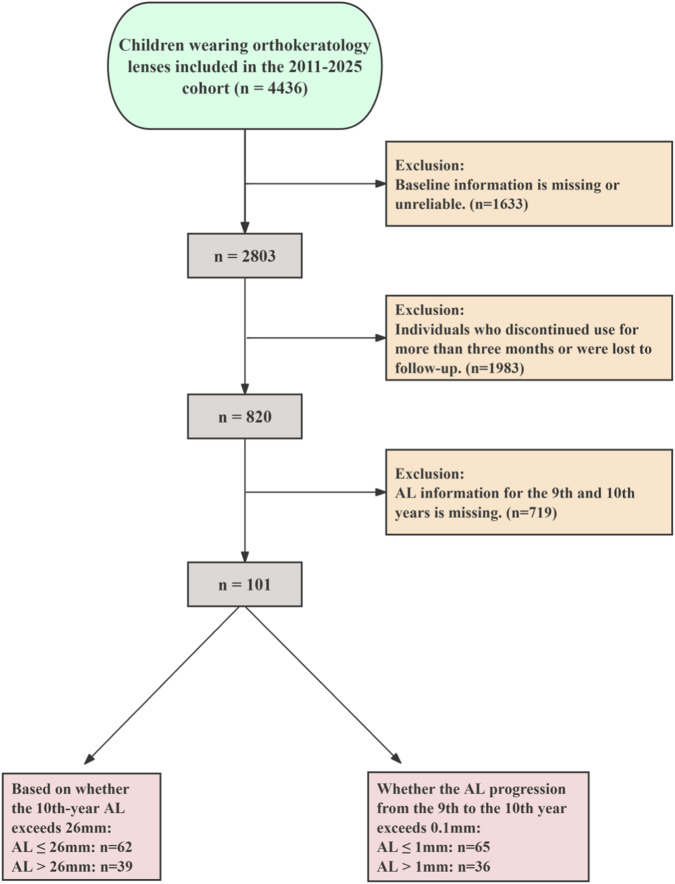
Flowchart of Inclusion and exclusion criteria for this study. AL, axial length.

We conducted a retrospective review of patients seen at Tianjin Medical University Eye Hospital between October 2011 and December 2025. The study was conducted in accordance with the tenets of the Declaration of Helsinki and approved by the Ethics Committee of Tianjin Medical University Eye Hospital (2024-KY67). All children and guardians have provided informed consent. The specific workflow is illustrated in Figure 1. Two primary outcomes were established to evaluate the long-term efficacy of Ortho-K treatment. The long-term outcome was defined as whether the AL exceeded 26 mm after 10 years of continuous wear, which is widely used as a structural marker associated with an increased risk of pathological myopia–related complications rather than a diagnostic criterion for pathological myopia itself. The short-term progression outcome was defined as rapid AL elongation, defined as an annual axial length increase greater than 0.1 mm between the 9th and 10th year of treatment. This threshold was selected as a conservative indicator of ongoing biological axial growth during a period when AL elongation is generally expected to slow or plateau, rather than as an established clinical diagnostic cutoff (Wu et al., 2025). Because both primary and secondary outcomes required precise axial length assessment at year 10 and between years 9 and 10, only participants with complete and continuous AL data at these predefined time points were eligible for inclusion. Patients lacking AL measurements at either year 9 or year 10 were excluded to avoid outcome misclassification. For baseline information on children, we included the following indicators: (1) Patient basic information: sex, age; (2) Refractive information: Cycloplegic Sphere Equivalent (SE), Cycloplegic Diopter Sphere (DS), Cycloplegic Diopter Cylindrical (DC), AL, keratometry parameters including: Steep Eccentricity (E), Flat E, Flat Keratometry (K), and Steep K, pupil distant (PD); (3) Corneal endothelium by relevant parameters: endothelial cell density (ECD), coefficient of variation in cell size (CV), and percentage of hexagonal cells (HEX). All ocular parameters were measured three times under cycloplegia by two experienced optometrists to obtain final values. The specific cycloplegia procedure is detailed in our previously published research (Hu et al., 2025). Exclusion criteria were as follows: (1) all subjects with missing baseline information or first 3 years AL data; (2) children with other ocular conditions such as strabismus, amblyopia, or ocular infections; (3) Children who received additional myopia control interventions alongside Ortho-K treatment (such as defocus spectacles, low-concentration atropine eye drops, repeated low-level red-light therapy and so on); (4) Children who discontinued Ortho-K lenses treatment or failed to adhere to prescribed wear protocols during treatment. Regarding the safety study of Ortho-K contact lens wear, we have disclosed this information in a separate study (Zhao et al., 2026).

Ortho-k lenses (Emerald Lenses; Euclid Systems Corp., Sterling, VA, oxygen permeability 95 × 10^−11^ (cm2/s) [mLO2/(mL·hPa)] @35 °C, geometric reverse four-arc surface) were used in this study. Subjects in the ortho-k group were provided with instructions on lens handling and care before receiving their lenses and were advised to wear them for 6–10 h per night. Ortho-k lenses were replaced during the follow-up period, with an average replacement interval of less than a year and a half. For the control group, subjects wore single-vision spectacles and continued to use them for 10 years or more without accepting any other treatments. Given diurnal variations in some values, subjects were required to undergo all tests approximately 2 h after their lenses were removed.

Cycloplegic autorefraction was obtained using an autorefractor (KR-800; Topcon, Japan), and then subjective optometry was performed on the following day to get the final refraction and to calculate the SE (SER was calculated as spherical plus 1/2 columnar). AL was measured without cycloplegia using a non-contact optical biometer (Lenstar LS900; Haag-Streit AG, Haag-Streit.com). AL was measured without a washout period, consistent with established Ortho-k lens protocols, as corneal epithelial remodeling induced by Ortho-K is minimal and has been shown not to materially affect AL assessment. Endothelial images were captured using a corneal endothelial cell counter (CEM-530, Beckman Coulter, United States).

#### Feature selection and model development

For both long-term outcomes (AL > 26 mm) and late-stage progression (Years 9th–10th), predictive models were developed using a training cohort and rigorously assessed in independent validation cohorts. The dataset was partitioned into training and validation sets (8:2) via 5-fold cross-validation to ensure model stability and reliability. Notably, the optimal cutoff values for first-year axial elongation (Change AL) as a predictor of late-stage progression were determined using the entire dataset to maximize statistical power and clinical representativeness. To identify the most significant predictors for both outcomes, three independent feature selection algorithms were employed on the training sets: Single-factor and Multifactor Logistic Regression, the Least Absolute Shrinkage and Selection Operator (LASSO) for dimension reduction, and the Boruta algorithm for all-relevant feature selection (Dai Z. et al., 2025) (Zhou et al., 2025).

### Statistical analysis

Continuous variables were presented as Mean ± SD or Median [IQR] based on normality tests, while categorical variables were expressed as frequencies and percentages. A significance threshold of p < 0.05 was applied. In the predictor selection process, variables that were significant (p < 0.05) in univariate logistic regression and passed collinearity diagnostics were retained for further analysis using multiple logistic regression. The discriminative performance of the models was evaluated using the Area Under the Receiver Operating Characteristic Curve (AUC) with 95% Confidence Intervals (CI). To provide a comprehensive assessment of diagnostic accuracy, we further calculated the sensitivity, specificity, and accuracy for each predictive model. Model calibration was assessed via calibration curves. To determine optimal clinical thresholds, the Youden index was used to identify the best cutoff values for Change AL in the first year across age groups (overall, 8–9 years, and 10–13 years). A dynamic nomogram and a web-based risk calculator were developed to facilitate clinical application.

## Results

### Baseline characteristics

Table 1 presents baseline information. A total of 101 eyes were included in this study, of which 62 had an AL ≤ 26 mm and 39 had an AL > 26 mm after 10 years. There were no significant differences in sex (p = 0.202, p > 0.05) or age (p = 0.384, p > 0.05) between the two groups. However, eyes that progressed to AL > 26 mm exhibited significantly higher baseline myopia (SE: −3.00 vs. −2.25 D, p = 0.001, p < 0.01) and significantly greater first-year AL change (Chang AL: 0.3 ± 0.1 vs. 0.1 ± 0.1 mm, p < 0.001).

**TABLE 1:** Baseline information table for different outcomes in this study.

Variable		Overall	AL ≤ 26 mm	AL > 26 mm	p
Eyes	​	101	62	39	​
Sex (%)	Female	61 (60.40)	41 (66.13)	20 (51.28)	0.202
​	Male	40 (39.60)	21 (33.87)	19 (48.72)	​
Age (median [IQR])	​	9.00 [8.00, 10.00]	9.00 [8.00, 10.75]	8.00 [8.00, 10.00]	0.384
SE (median [IQR])	​	−2.50 [-3.25, −1.75]	−2.25 [-2.75, −1.50]	−3.00 [-4.50, −2.00]	0.001
DS (median [IQR])	​	−2.25 [-3.00, −1.75]	−2.00 [-2.75, −1.50]	−3.00 [-4.25, −2.00]	0.002
DC (median [IQR])	​	−0.50 [-0.75, 0.00]	0.00 [-0.50, 0.00]	−0.50 [-1.00, −0.25]	<0.001
Flat E (median [IQR])	​	0.57 [0.50, 0.63]	0.56 [0.49, 0.60]	0.62 [0.54, 0.66]	0.001
Steep E (mean (SD))	​	0.56 (0.14)	0.55 (0.13)	0.59 (0.16)	0.131
Flat K (mean (SD))	​	43.26 (1.02)	43.33 (0.99)	43.16 (1.07)	0.414
Steep K (median [IQR])	​	44.13 [43.54, 45.12]	44.12 [43.46, 44.88]	44.13 [43.63, 45.28]	0.451
Pupil (mean (SD))	​	5.07 (0.95)	4.94 (0.94)	5.28 (0.96)	0.084
AL (median [IQR])	​	24.49 [23.76, 24.77]	24.09 [23.68, 24.69]	24.67 [24.45, 25.42]	0.001
ECD (mean (SD))	​	3,165.69 (197.08)	3,202.36 (171.45)	3,107.40 (222.13)	0.018
CV (median [IQR])	​	0.25 [0.23, 0.27]	0.25 [0.23, 0.28]	0.25 [0.23, 0.26]	0.599
HEX (median [IQR])	​	0.76 [0.70, 0.81]	0.77 [0.71, 0.82]	0.76 [0.69, 0.80]	0.509
Change AL (median [IQR])	​	0.21 [0.12, 0.30]	0.16 [0.10, 0.23]	0.31 [0.23, 0.34]	<0.001
AL 10 years (median [IQR])	​	25.67 [25.17, 26.46]	25.34 [24.96, 25.61]	26.63 [26.28, 27.04]	<0.001

Variable		Overall	AL ≤ 1 mm	AL > 1 mm	p
Eyes	​	101	65	36	​
Sex (%)	Female	61 (60.40)	41 (63.08)	20 (55.56)	0.598
​	Male	40 (39.60)	24 (36.92)	16 (44.44)	​
Age (median [IQR])	​	9.00 [8.00, 10.00]	9.00 [8.00, 11.00]	8.00 [8.00, 9.00]	0.007
SE (median [IQR])	​	−2.50 [-3.25, −1.75]	−2.50 [-3.25, −1.75]	−2.38 [-3.25, −1.69]	0.675
DS (median [IQR])	​	−2.25 [-3.00, −1.75]	−2.50 [-3.00, −1.75]	−2.00 [-3.00, −1.44]	0.475
DC (median [IQR])	​	−0.50 [-0.75, 0.00]	0.00 [-0.75, 0.00]	−0.50 [-1.00, 0.00]	0.132
Flat E (median [IQR])	​	0.57 [0.50, 0.63]	0.54 [0.49, 0.61]	0.60 [0.56, 0.66]	0.01
Steep E (mean (SD))	​	0.56 (0.14)	0.55 (0.15)	0.59 (0.13)	0.223
Flat K (mean (SD))	​	43.26 (1.02)	43.32 (1.04)	43.15 (0.99)	0.416
Steep K (median [IQR])	​	44.13 [43.54, 45.12]	44.14 [43.60, 45.08]	44.02 [43.52, 45.16]	0.799
Pupil (mean (SD))	​	5.07 (0.95)	5.03 (0.88)	5.14 (1.08)	0.572
AL (median [IQR])	​	24.49 [23.76, 24.77]	24.56 [23.80, 24.90]	24.45 [23.67, 24.65]	0.203
ECD (mean (SD))	​	3,165.69 (197.08)	3,199.44 (199.17)	3,104.75 (180.27)	0.02
CV (median [IQR])	​	0.25 [0.23, 0.27]	0.25 [0.23, 0.29]	0.24 [0.23, 0.26]	0.114
HEX (median [IQR])	​	0.76 [0.70, 0.81]	0.76 [0.69, 0.82]	0.76 [0.72, 0.80]	0.994
Change AL (median [IQR])	​	0.21 [0.12, 0.30]	0.14 [0.08, 0.21]	0.32 [0.30, 0.36]	<0.001

IQR, interquartile range; SD, standard deviation; AL, axial length; ECD, endothelial cell density; CV, coefficient of variation in cell size; HEX, hexagonal cells; DS, diopter sphere; DC, diopter cylindrical; SE, sphere equivalent.

### Feature selection for AL outcomes

Univariable and multivariable logistic regression analyses were conducted to identify key predictors for long-term and late-stage AL outcomes. For the 10-year outcome (AL > 26 mm), univariable analysis revealed that baseline SE, DS, Flat E, baseline AL, and first-year AL change were significantly associated with the outcome (p < 0.05). Subsequent multivariable analysis confirmed that baseline AL and Change AL (p < 0.05) were independent predictors of AL exceeding 26 mm after a decade of treatment. Regarding late-stage progression (annual AL increase >0.1 mm between years 9 and 10), univariable analysis identified age, ECD, Flat E, and Change AL as significant factors (all p < 0.05). In the multivariable model, change in AL emerged as the most independent predictor for rapid late-stage progression (p < 0.01). Table S presents P-values for single-factor and multiple-factor regression analyses.

**FIGURE 2:**
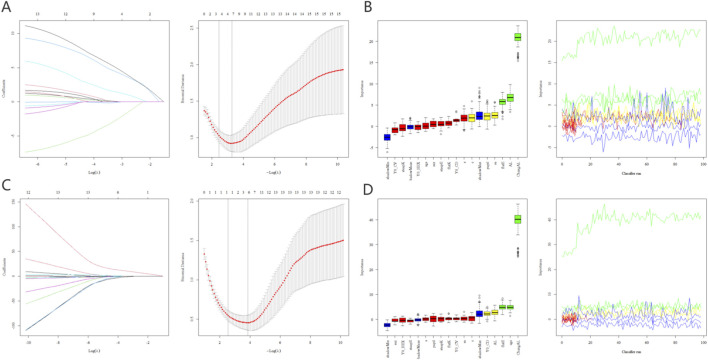
Feature selection for AL outcomes using LASSO and Boruta algorithms. Figure **(A,B)** illustrate the identification of key predictors for the long-term risk of high myopia (defined as AL > 26 mm after 10 years of Ortho-K lenses wear) via LASSO regression coefficient profiles and Boruta variable importance rankings, respectively. Figure **(C,D)** present the feature selection results for predicting rapid short-term progression (defined as an annual AL increase >0.1 mm between the 9th and 10th year of treatment), utilizing LASSO-based dimension reduction and Boruta’s all-relevant feature analysis to determine the most significant contributing variables. LASSO, Least Absolute Shrinkage and Selection Operator; Ortho-K lenses, Orthokeratology Lenses; AL, axial length.

The LASSO and Boruta algorithms successfully identified key predictors for both long-term and short-term outcomes. For the 10-year high myopia risk (AL > 26 mm), predictors were screened and ranked by importance (Figures 2A,B). Similarly, for the late-stage rapid progression (9th-10th year progression >0.1 mm), relevant features were selected using the same robust pipeline (Figures 2C,D). In the 10-year AL outcome, LASSO selected four variables: SE, Flat E, base AL, and change AL. Boruta selected three variables: Flat E, base AL, and change AL. In the progression from Year 9 to Year 10 AL outcomes, LASSO selected the variable Change AL, while Boruta selected three variables: Change AL, age, and Flat E.

### Model performance for predicting AL > 26 mm in 10 years

**FIGURE 3:**
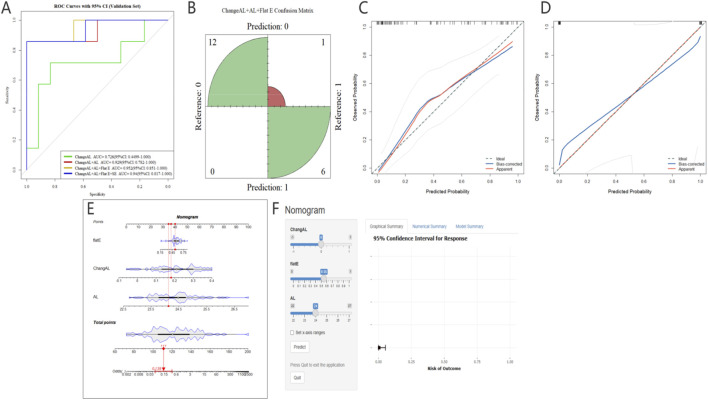
Development, validation, and clinical application of the predictive model for long-term axial length (AL) outcomes. Figure **(A)** displays the ROC curves in the independent validation cohort, comparing the discriminative performance of models with different variable combinations for predicting high myopia risk (AL > 26 mm) after 10 years of Ortho-K treatment. The classification accuracy of the optimized model is further visualized in the confusion matrix **(B)**, showing the distribution of prediction results in the validation set. Calibration curves for the training **(C)** and validation **(D)** cohorts demonstrate high consistency between the predicted probabilities and actual clinical observations. To facilitate clinical utility, a dynamic nomogram **(E)** was established for individualized risk assessment, complemented by a web-based risk calculator interface **(F)** designed for rapid screening and decision-making in long-term pediatric Ortho-K management. Ortho-K: Orthokeratology; ROC, Receiver Operating Characteristic Curve; AL, axial length.

Model performance across different variable combinations is summarized in Table 2. The model, including Change AL, demonstrated modest discriminative ability (AUC = 0.726). Incorporation of baseline AL markedly improved prediction performance, and further inclusion of corneal flat E resulted in substantial gains. The Change AL + AL + Flat E model achieved an AUC of 0.952 (95% CI: 0.851–1.000), with high sensitivity (0.857), specificity (1.000), and overall accuracy (0.947). Adding SE did not further improve discrimination, indicating a limited incremental benefit beyond axial and corneal parameters. Model validation results are illustrated in Figure 3. In the independent validation cohort, ROC curve analysis (Figure 3A) confirmed excellent discriminative performance of the optimized model for identifying children at risk of developing high myopia after 10 years of Ortho-K treatment. The confusion matrix (Figure 3B) demonstrated a high classification accuracy with minimal misclassification. Calibration analysis showed good agreement between predicted and observed risks in both the training (Figure 3C) and validation (Figure 3D) cohorts, indicating robust calibration across datasets. To enhance clinical applicability, a nomogram (Figure 3E) was constructed from the final multivariable model, enabling individualized risk estimation. In addition, a dynamic web-based risk calculator (Figure 3F) was developed to facilitate rapid clinical screening and decision-making in long-term pediatric Ortho-K management.

**TABLE 2:** Performance parameters of each model when different variables are included for different outcomes.

Variable	AUC	95% CI lower	95% CI upper	Sensitivity	Specificity	Accuracy	Youden index
Based on whether AL is > 26 mm for the outcome							
Change AL	0.726	0.449	1	0.714	0.833	0.789	0.547
Change AL + AL	0.929	0.782	1	0.857	1	0.947	0.857
Change AL + AL + flat E	0.952	0.851	1	0.857	1	0.947	0.857
Change AL + AL + flat E + SE	0.94	0.817	1	0.857	1	0.947	0.857
Based on whether AL is > 1 mm for the outcome							
Change AL	0.995	0.979	1	1	0.923	0.95	0.923
Change AL + age + flat E	1	1	1	1	1	1	1

AL, axial length.

### Model performance for predicting late-stage rapid AL progression (>0.1 mm/year)

For the second outcome, defined as rapid late-stage axial elongation (AL increase >0.1 mm between the 9th and 10th year of Ortho-K wear), model performance is also presented in Table 2. The model based on Change AL demonstrated good discriminative performance within this cohort in the test set (AUC = 0.995, 95% CI: 0.979–1.000), with high sensitivity, specificity, and accuracy. The inclusion of age and Flat E further improved classification performance, achieving an AUC of 1.000, indicating a good ability to identify children at risk of late-stage AL progression.

**FIGURE 4:**
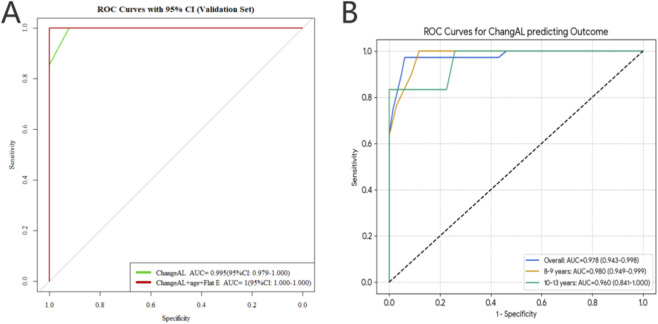
Performance evaluation and optimal cutoff determination for predicting late-stage AL progression. Figure **(A)** presents the ROC curves of the predictive model in the independent validation cohort, demonstrating its discriminative capability for identifying rapid AL progression (defined as an annual increase >0.1 mm between the 9th and 10th year of Ortho-K wear). To enhance clinical applicability across different developmental stages, Figure **(B)** illustrates the determination of optimal Change AL in the first year cutoff values derived from the entire dataset, categorized by age groups. These cutoff points were identified using the maximum Youden index to provide evidence-based thresholds for monitoring AL changes and tailoring intervention strategies for children of varying ages during long-term treatment. Ortho-K:Orthokeratology; ROC, Receiver Operating Characteristic Curve; AL, axial length.

As shown in Figure 4A, ROC analysis in the independent test set confirmed the strong discriminative capability of the predictive model for detecting rapid AL progression. To establish clinically meaningful thresholds, Figure 4B presents ROC curves derived from the entire dataset, stratified by age groups. Analysis of the whole dataset revealed that the predictive value of first-year Change AL varied by age. For the overall population, the optimal cutoff for predicting rapid progression was 0.28 mm (AUC = 0.978). In the age-specific analysis, the 8–9 years group showed a cutoff of 0.28 mm (Sensitivity: 100%, Specificity: 88.2%), while the 10–13 years group had a slightly higher cutoff of 0.29 mm (Sensitivity: 83.3%, Specificity: 100%) (Table 2; Figure 4). Optimal cutoff values for first-year Change AL were determined using the maximum Youden index, providing evidence-based thresholds for monitoring late-stage AL progression and guiding individualized intervention strategies across different developmental stages (Table 3).

**TABLE 3:** Cutoff values for first-year AL change in children of different age groups for predicting stable AL progression in the tenth year.

Group	AUC (95% CI)	Cutoff	Sensitivity	Specificity	Youden index
Overall	0.978 (0.943–0.998)	0.28	0.972	0.938	0.911
8–9 years	0.980 (0.949–0.999)	0.28	1	0.882	0.882
10–13 years	0.960 (0.841–1.000)	0.29	0.833	1	0.833

## Discussion

To our knowledge, this is the first study to predict AL progression after 10 years of Ortho-K lens treatment using baseline refractive data and early changes in AL. Addressing a practical clinical question, we focused on outcomes of AL progression control in adulthood and final AL status. The first-year change in AL demonstrated a strong association with both long-term outcomes in this cohort. Additionally, baseline AL and Flat E demonstrate greater significance than other values in determining the final AL. With the increasing prevalence and diversity of combined control approaches over the past few years, options have gradually expanded (Zhang et al., 2025) (Cao et al., 2025) (Xiong et al., 2024) (Liu G. et al., 2025). This study provides preliminary evidence that early AL responses to Ortho-K lens may help inform future research on risk stratification and the potential role of adjunctive myopia control strategies.

It is important to interpret the defined outcomes in the appropriate clinical context. In this study, AL exceeding 26 mm was used as a structural risk marker associated with an increased likelihood of pathological myopia–related complications, rather than as a diagnostic threshold for pathological myopia itself. Likewise, the secondary outcome of late-stage axial length progression (>0.1 mm between years 9 and 10) was intended to capture ongoing biological axial activity during a period when axial elongation is generally expected to decelerate. These outcome definitions were therefore designed to reflect relative risk stratification and late-stage growth dynamics, rather than definitive clinical endpoints.

Dynamic AL is undoubtedly a significant predictor in this study. Ruan et al. predicted short-term AL control efficacy based on AL changes after 1 month of Ortho-K lens wear, finding that short-term AL changes better reflect individual responsiveness to Ortho-K lens treatment than other baseline factors (Ruan et al., 2025). Our preliminary machine-learning research on Ortho-K lens wear data over the first 5 years yielded similar findings. Compared with other baseline data, the change in axial length during the first year proved a better predictive indicator (Wang et al., 2026a) (Wang et al., 2026b). Ortho-K lenses alter the shape of the cornea’s anterior surface, creating a myopic defocus in the peripheral retina. The AL changes observed during the first year reflect the eye’s “initial sensitivity” to this biomechanical stimulus. Research has found that after wearing Ortho-K lenses, the choroid thickens rapidly (Wu et al., 2023). Liu et al. found that dynamic changes in the choroidal vasculature provide a possible mechanism underlying the efficacy of myopia control via ortho-k (Liu M. et al., 2025). The above possible mechanisms are also consistent with the predictive significance of dynamic changes in AL. If AL control is well maintained in the first year, it indicates that the individual is highly sensitive to the defocus signal generated by Ortho-K lenses and possesses a strong choroidal compensatory capacity. This sensitivity may persist over time, potentially contributing to reduced mechanical tensile stress on the sclera in subsequent years.

Additionally, from a developmental perspective, AL growth is essentially a controlled expansion of the sclera. The data from the first year reflects the “inertia” of ocular development. Development tends toward homeostasis (Hu et al., 2023). Once a favorable “myopic defocus signal balance” is established during the first year through Ortho-K lenses treatment, the degradation and synthesis of scleral fibers often maintain a dynamic equilibrium at a low level, thereby potentially contributing to a reduced risk of high myopia in adulthood. Adolescence is the most active stage of eye development. The response to orthokeratology lenses during the first year essentially tests the plasticity of an individual’s developmental gene expression in response to environmental interventions (Wal et al., 2004).

Another secondary parameter we identified is the baseline Flat E. A higher baseline Flat E typically indicates that the cornea flattens more rapidly from the center to the periphery. The design principle of Ortho-K lenses involves redistributing the cornea through reverse geometry. A higher baseline E-value is associated with a steeper peripheral corneal slope. When the lens flattens the central area, the mid-periphery is more prone to physical “tissue accumulation.” This geometry facilitates the formation of a steeper defocus ring (Treatment Zone Ring) on the cornea. A steeper defocus ring delivers a stronger myopic defocus signal, more effectively triggering retinal-scleral steady-state regulation and inhibiting AL growth (Atchison and Charman, 2024). Additionally, research has found that myopia control requires peripheral defocus to reach a certain threshold. Individuals with a larger baseline Flat E typically experience more pronounced changes in corneal curvature in the mid-periphery when wearing Ortho-K lenses with identical parameters (Swiatczak et al., 2024). In this study, Flat E was the second most significant predictor of AL change after the first year. Future studies with larger sample sizes are warranted to further clarify its role in treatment outcomes.

Previous studies have indicated that age influences the treatment outcomes of Ortho-K lenses (Grytz and El Hamdaoui, 2017) (Yu and Zhou, 2022). T. M. et al. found no statistically significant differences in treatment outcomes or safety of Ortho-K lenses, regardless of initial age (Jakobsen et al., 2025). To investigate the long-term treatment outcomes of Ortho-K lenses among children of different age groups, we conducted an age-stratified analysis of the entire dataset. The high AUC values observed across all age groups (0.960–0.980) underscore that the first-year AL change is not merely a short-term outcome but a good early indicator of long-term AL growth patterns. From a developmental perspective, the 0.28 mm cutoff for the 8–9 years group serves as a critical “success threshold” during the peak of the childhood growth spurt. In younger cohorts, characterized by heightened scleral plasticity and robust developmental growth, a first-year AL increase below the 0.28 mm threshold signifies that peripheral myopic defocus has effectively superseded pro-myopic elongation signals. This suggests an early and successful recalibration of the emmetropization feedback loop, establishing a homeostatic steady state that mitigates long-term axial progression. From a clinical research perspective, the first year of Ortho-K lenses may function as an informative observation period for assessing individual ocular biomechanical responsiveness. AL progression within these cutoffs indicates a stabilized homeostatic state, likely mediated by choroidal thickening and attenuated scleral creep, and may be associated with sustained AL control over longer follow-up periods. Conversely, exceeding these thresholds may indicate a higher-risk growth pattern, suggesting that closer monitoring or evaluation of adjunctive interventions could be considered in future prospective studies.

To explore the potential translational value of these longitudinal findings, we developed a risk nomogram and a digital calculator designed to estimate the probability that the final AL will exceed the 26 mm high-myopia threshold. By integrating baseline parameters—specifically, baseline AL and flat E—with the first-year Change in AL, the model provides an individualized risk estimate within this study cohort. The practical utility of this nomogram lies in its ability to quantify long-term risk during the earliest stages of intervention. For instance, a higher baseline flat E contributes to a more favorable point score, reflecting its role in generating robust peripheral myopic defocus, whereas a first-year Change AL exceeding the 0.28–0.29 mm cutoff significantly escalates the risk of surpassing 26 mm by adulthood. This visual and computational tool allows clinicians to move beyond a “one-size-fits-all” approach, which may help generate hypotheses regarding early identification of individuals who could benefit from closer follow-up or adjunctive interventions in future clinical trials. Ultimately, this nomogram illustrates how early biometric data might be leveraged for long-term risk estimation in research settings, highlighting the importance of the first year as a potential window for future prospective intervention studies.

Long-term attrition represents an important source of potential selection bias in this study. Children who completed 10 years of orthokeratology treatment and follow-up are likely to differ systematically from those who discontinued treatment or were lost to follow-up, potentially exhibiting higher adherence, more favorable early treatment responses, or different family and socioeconomic characteristics. As a result, the estimated associations and predictive performance may not be fully generalizable to all children initiating orthokeratology treatment, and the external validity of the findings should be interpreted with caution.

This study also has other limitations. First, as a retrospective cohort study, it spans a long timeframe but ultimately incorporates a relatively small dataset. In the future, we plan to collaborate with multiple centers to collect more relevant sample data. Second, the variables included in this study are relatively traditional, which is related to the time span. With technological advancements, Ortho-K lenses are also undergoing continuous improvements. Moving forward, we will establish a cohort with more comprehensive baseline data to provide evidence-based contributions to the long-term efficacy of myopia control. Third, the predictive model employed is traditional, constrained by limitations in sample size. The extremely large odds ratios and near-ceiling AUC values observed in some models likely reflect the combined effects of limited sample size, strong correlations among predictors, and low event rates. These conditions increase the risk of model overfitting and coefficient instability, even with internal validation. Consequently, the reported model performance should be regarded as exploratory and hypothesis-generating rather than evidence of definitive clinical prediction accuracy. Upon expanding the sample size, we will supplement the model using additional machine learning methods. In addition, a formal baseline comparison between included and excluded participants could not be performed. Baseline data for many excluded individuals were incomplete or inconsistently recorded, particularly for corneal and endothelial parameters, which precluded reliable statistical comparison. This limitation prevents a direct assessment of cohort representativeness.

## Conclusion

This 10-year longitudinal study suggests that first-year AL change may serve as an early indicator of long-term AL growth trajectories in children wearing orthokeratology lenses. By integrating early axial elongation with baseline ocular parameters, the proposed models may help identify children at higher risk of unfavorable long-term outcomes. However, given the retrospective design, limited sample size, and potential selection bias, these findings should be considered hypothesis-generating. Prospective, multicenter studies with external validation are required before these models can be applied to routine clinical decision-making.

## Data Availability

The raw data supporting the conclusions of this article will be made available by the authors, without undue reservation.
