# Supplementary material for: Association between first-year axial elongation and 10-year myopia progression in children wearing orthokeratology lenses: a ten-year longitudinal study
Source: Front Cell Dev Biol. 2026 Mar 13;14:1786621. doi: 10.3389/fcell.2026.1786621 (PMC13021867; doi:10.3389/fcell.2026.1786621)
Supplement: Supplementary file 1 [file Table1.docx]

Table S. Results of univariate and multivariate logistic regression for different outcomes in the training set.

| **Variable** |  | **AL ≤ 26mm**  **(N=50)** | **AL > 26mm**  **(N=32)** | **OR (univariable)** | **OR (multivariable)** |
| --- | --- | --- | --- | --- | --- |
| Sex | Female | 35 (70%) | 17 (53.1%) |  |  |
|  | Male | 15 (30%) | 15 (46.9%) | 2.06 (0.82-5.17, p=.124) |  |
| Age (Mean ± SD) |  | 9.5 ± 1.8 | 8.9 ± 1.3 | 0.78 (0.58-1.06, p=.117) |  |
| SE (Mean ± SD) |  | -2.4 ± 1.2 | -3.3 ± 1.7 | 0.63 (0.45-0.90, p=.011) | 0.18 (0.02-2.05, p=.168) |
| DS (Mean ± SD) |  | -2.2 ± 1.1 | -3.0 ± 1.6 | 0.63 (0.43-0.93, p=.018) | 2.33 (0.19-28.80, p=.508) |
| DC (Mean ± SD) |  | -0.4 ± 0.6 | -0.6 ± 0.6 | 0.50 (0.23-1.09, p=.081) |  |
| Flat E (Mean ± SD) |  | 0.5 ± 0.1 | 0.6 ± 0.1 | 1252.55 (6.48-242028.00, p=.008) | 100.48 (0.15-67117.02, p=.165) |
| Steep E (Mean ± SD) |  | 0.5 ± 0.1 | 0.6 ± 0.1 | 6.22 (0.22-175.78, p=.283) |  |
| Flat K (Mean ± SD) |  | 43.4 ± 1.0 | 43.1 ± 1.0 | 0.77 (0.49-1.21, p=.260) |  |
| Steep K (Mean ± SD) |  | 44.4 ± 1.2 | 44.3 ± 1.1 | 0.98 (0.66-1.46, p=.926) |  |
| Pupil (Mean ± SD) |  | 4.9 ± 0.9 | 5.3 ± 1.0 | 1.55 (0.97-2.48, p=.064) |  |
| AL (Mean ± SD) |  | 24.2 ± 0.7 | 24.8 ± 0.8 | 3.07 (1.51-6.26, p=.002) | 6.04 (1.52-23.91, p=.010) |
| DCD (Mean ± SD) |  | 3197.9 ± 172.2 | 3132.2 ± 208.7 | 1.00 (1.00-1.00, p=.127) |  |
| CV (Mean ± SD) |  | 0.3 ± 0.0 | 0.2 ± 0.0 | 0.03 (0.00-1235.30, p=.523) |  |
| HEX (Mean ± SD) |  | 0.8 ± 0.1 | 0.7 ± 0.1 | 0.13 (0.00-12.95, p=.387) |  |
| Sex | Female | 31 (59.6%) | 16 (55.2%) |  |  |
|  | Male | 21 (40.4%) | 13 (44.8%) | 1.20 (0.48-3.00, p=.698) |  |
| Age (Mean ± SD) |  | 10.0 ± 1.9 | 8.7 ± 1.0 | 0.55 (0.37-0.80, p=.002) | 2.98 (0.77-11.52, p=.114) |
| SE (Mean ± SD) |  | -2.9 ± 1.5 | -2.6 ± 1.8 | 1.13 (0.84-1.51, p=.434) |  |
| DS (Mean ± SD) |  | -2.6 ± 1.3 | -2.4 ± 1.7 | 1.14 (0.81-1.59, p=.451) |  |
| DC (Mean ± SD) |  | -0.6 ± 0.8 | -0.5 ± 0.5 | 1.24 (0.62-2.50, p=.542) |  |
| Flat E (Mean ± SD) |  | 0.5 ± 0.1 | 0.6 ± 0.1 | 63.74 (1.00-4067.08, p=.050) |  |
| Steep E (Mean ± SD) |  | 0.6 ± 0.2 | 0.6 ± 0.1 | 3.82 (0.16-93.11, p=.411) |  |
| Flat K (Mean ± SD) |  | 43.4 ± 0.9 | 43.2 ± 1.0 | 0.84 (0.51-1.39, p=.498) |  |
| Steep K (Mean ± SD) |  | 44.6 ± 1.2 | 44.3 ± 1.1 | 0.81 (0.54-1.23, p=.325) |  |
| Pupil (Mean ± SD) |  | 5.0 ± 0.9 | 5.1 ± 1.1 | 1.18 (0.73-1.91, p=.495) |  |
| AL (Mean ± SD) |  | 24.5 ± 0.8 | 24.2 ± 0.9 | 0.71 (0.40-1.27, p=.251) |  |
| DCD (Mean ± SD) |  | 3222.9 ± 204.6 | 3128.6 ± 186.0 | 1.00 (1.00-1.00, p=.048) | 1.00 (0.99-1.00, p=.171) |
| CV (Mean ± SD) |  | 0.3 ± 0.0 | 0.2 ± 0.0 | 0.00 (0.00-8.36, p=.108) |  |
| HEX (Mean ± SD) |  | 0.8 ± 0.1 | 0.8 ± 0.1 | 0.47 (0.00-47.53, p=.747) |  |

SD: Standard Deviation; AL: axial length; ECD: endothelial cell density; CV: coefficient of variation in cell size; HEX: hexagonal cells; DS: Diopter Sphere; DC: Diopter Cylindrical; SE: Sphere Equivalent.
